# Update on pathogenesis and diagnosis flow of normoalbuminuric diabetes with renal insufficiency

**DOI:** 10.1186/s40001-021-00612-9

**Published:** 2021-12-11

**Authors:** Le Deng, Wenjie Li, Gaosi Xu

**Affiliations:** Department of Nephrology, The Second Affiliated Hospital of Nanchang University, No. 1, Minde Road, Donghu District, Nanchang, Jiangxi 330006 People’s Republic of China

**Keywords:** Diabetes Mellitus, Normoalbuminuric, Phenotype, Glomerular Filtration Rate, Renal Insufficiency

## Abstract

In recent decades, the prevalence of diabetic kidney disease has remained stable and appears to be a wide heterogeneity. Normoalbuminuric diabetes with renal insufficiency, which is characterized by a decline in the glomerular filtration rate in the absence of albuminuria, has been identified as an albuminuria-independent phenotype of diabetic kidney disease. Epidemiological data demonstrate that normoalbuminuric phenotype is prevalent. Compared to albuminuric phenotype, normoalbuminuric phenotype has distinct clinical characteristics and a wide heterogeneity of pathological features. Currently, the pathogenesis of normoalbuminuric phenotype remains unclear. Additionally, the flow of diagnosing normoalbuminuric phenotype is not perfect. In this article, we review the latest studies addressing the epidemiology, clinical characteristics, and pathology of normoalbuminuric phenotype. Based on the studies of clinical features and renal histopathologic changes, we attempt to propose an underlying pathogenesis model and a flow chart for diagnosing normoalbuminuric phenotype.

## Introduction

Over the years, albuminuria has been regarded as a pivotal indicator of diabetic kidney disease (DKD) and invariably precedes estimated glomerular filtration rate (eGFR) loss. However, this classical view has been challenged by evidence that a considerable proportion of patients with diabetes have renal insufficiency in the absence of albuminuria, referred to normoalbuminuric diabetic kidney disease [1, 2] or nonalbuminuric diabetic nephropathy [1, 3] or diabetic kidney disease without albuminuria [4], or nonalbuminuric renal insufficiency in type 2 diabetes [5], defined as eGFR < 60 mL/min per 1.73 m^2^ and in the absence of albuminuria (urinary albumin excretion rate (UAER) < 30 mg/24 h or urinary albumin creatinine ratio (UACR) < 30 mg/g), has been identified as albuminuria-independent phenotype of DKD [6, 7]. The Italian Diabetes Society and Italian Society of Nephrology also well described the nonalbuminuric renal impairment phenotype in a joint position statement on the natural history of diabetes mellitus in 2020 [8].

The combination of renal insufficiency and normoalbuminuria in diabetes was first highlighted by Lane et al. in 1992 [9], and has been confirmed for decades. The prevalence of the normoalbuminuric diabetes with renal insufficiency (NADRI) increased and has become a prevailing phenotype of DKD at present [10]. Even though these patients carry a lower risk for chronic kidney disease (CKD) progression, end-stage renal disease, or cardiovascular disease or all-cause mortality compared to those with albuminuric diabetes with renal insufficiency (ADRI) [7, 11–19], it significantly contributes to the burden of advanced CKD or end-stage renal disease morbidity [6, 13, 20]. Moreover, mortality rates generally increased in these patients [10].

Currently, the natural course and potential pathogenesis of NADRI are still elusive. In this article, we review the latest studies addressing the epidemiology, clinical characteristics, and pathology of NADRI. Based on the studies of clinical features and renal histopathologic changes, we attempt to propose an underlying pathogenesis model and a flow chart for diagnosing NADRI.

## Epidemiology

Two early studies reported that renal insufficiency may occur in both type 1 diabetes mellitus (T1DM) and type 2 diabetes mellitus (T2DM) with normoalbuminuria [9, 21], and these observations have been confirmed in subsequent studies.

In T2DM, the prevalence of NADRI ranges from 14 to 57% in cross-sectional studies, with differences among surveys depending on the geographic area and the calculation formula of eGFR [5, 13, 14, 21–36]. A higher incidence of NADRI (more than 60%) was observed in recent years [37–41]. Similar high prevalence was noted in multicenter multinational interventional researches, in which values were affected by different inclusion criteria, nevertheless [42–45]. Different from T2DM, the prevalence variation of NADRI in T1DM is significant, which ranges from 2 to 58% [9, 15, 38, 46–48]. A recent study indicated that the proportion of NADRI was remarkably high (58.6%) [49].

These data are in line with the increasing prevalence of declined eGFR and the decreasing incidence of albuminuria observed in diabetes [50, 51]. The increasing prevalence of NADRI may be partly due to the wide application of renin–angiotensin–aldosterone system (RAAS) inhibitors, advances in the treatment of antihyperglycemic, antihypertensive and hypolipidemic, and smoking cessation [19, 24, 52–54].

## Clinical characteristics

The reported clinical features of NADRI include a higher proportion of females [7, 15, 16, 36, 37, 47], more likely to be older [15, 16, 47] and nonsmokers [7, 15, 16, 36, 37, 47]; a shorter diabetes duration [36, 55, 56]; lower levels of systolic blood pressure [7, 13, 15, 16, 36, 37, 47, 55, 56], diastolic blood pressure [13, 15, 16, 47, 55], hemoglobin A1c (HbA1c) [7, 15, 36, 37, 47], triglycerides[15, 36, 37, 47], total cholesterol [13, 15, 47, 55], and low-density lipoprotein cholesterol [13, 15, 47]; higher levels of high-density lipoprotein cholesterol [15, 18, 36, 47]; and lower prevalence of diabetic retinopathy [7, 15, 18, 37, 55, 56], neuropathy [55], and cardiovascular disease [7, 16, 37], as compared to patients with ADRI (Table 1).

**Table 1:** Comparison of NADRI with ADRI in clinical and laboratory characteristics

Item	Rigalleau 2007[18]	Afghahi 2013[37]	Shimizu 2013 [56]	De cosmo 2014[36]	Thorn 2015[47]	Penno 2018[7]	Lamacchia 2018[15]	Yamanouchi 2019[13]	Buyadaa 2020[16]	Dai 2021 [55]
*N*	15/74	10,111/6211	15/139	13,660/14,684	78/424	1476/1230	719/676	82/164	432/345	45/95
Age (years)	68 ± 9/64 ± 12	2.16 ± 2.10/1.99 ± 1.23	62.5 ± 6.2/62.1 ± 9.6	74.0 ± 8.0/73.4 ± 8.5	55.1(45.5, 61.3)/43.7(36.1, 50.7) *	74.4 ± 8.1/73.2 ± 9.1	66 ± 12/60 ± 14*	63(56, 67)/64(56, 70)	66.7(62.4, 72)/66.0(62.1, 71.2) *	67.6 ± 12.2/66.2 ± 12.1
Female (%)	66/40	64/42*	53.3/34.5	57.8/40.5*	77/42*	60.23/37.72*	65/41.5*	34/32	58.4/50.2*	48.9/42.1
BMI (kg/m^2^)	27.0 ± 4.5/26.9 ± 4.3	29.3 ± 5.2/29.5 ± 5.3*	22.2 ± 2.2/23.5 ± 3.4	29.7 ± 5.1/30.0 ± 5.2	25.2 ± 3.3/25.9 ± 4.1	29.06 ± 5.12/29.14 ± 5.05	26.2 ± 4.5/26.5 ± 4.9	23(21, 25)/24(22, 26)	32.4 ± 5.4/32.7 ± 5.7	25.4(23.2, 26.8)/24.2(22.3, 25.7)
Smoking (%)	20/52	6.1/8.0*	–	8.8/13.4*	12.0/24.0*	36.65/46.75*	13.5/24.3*	63/61	6/12.7*	20/29.5
HTN (%)	–	–	–	–	–	92.89/95.04	53.9/61.9	-	-	75.6/88.4
SBP (mmHg)	143 ± 16/147 ± 19	136 ± 17/139 ± 19*	129.3 ± 14.3/146.9 ± 21.0*	139.5 ± 18.9/143.8 ± 20.5*	137 ± 20/147 ± 21*	138.6 ± 18.7/140.9 ± 19.6*	137 ± 20/141 ± 20*	130(120, 145)/146(134, 162)*	133.0 ± 17.0/144.8 ± 18.8*	137 ± 20/152 ± 22*
DBP(mmHg)	79 ± 8/81 ± 10	73 ± 9.8/74 ± 10.3	75.6 ± 10.0/76.8 ± 12.4	77.3 ± 9.8/78.5 ± 10.3	75 ± 9/82 ± 11*	77.4 ± 9.7/77.6 ± 10.1	76 ± 10/78 ± 10*	75(68, 80)/80(70, 90)*	70.8 ± 10.6/72.6 ± 12.0*	79 ± 12/85 ± 12*
HbA1c (%)	9.0 ± 1.3/8.5 ± 1.6	6.97 ± 3.16/7.25 ± 3.38*	8.3 ± 2.2/7.4 ± 2.0	7.4 ± 1.4/7.7 ± 1.5*	8.1 ± 1.2/9.0 ± 1.5*	7.58 ± 1.45/7.85 ± 1.65*	8.0 ± 1.5/8.3 ± 1.7*	7.2(6.5, 9.0)/6.9(6.0, 8.3)	8.1 ± 1.0/8.3 ± 1.0	8.3 ± 2.5/9.0 ± 2.3
DD (years)	14 ± 5/19 ± 11	10.0 ± 7.7/13.0 ± 8.2	7.4 ± 6.4/13.1 ± 8.8*	13.3 ± 10.4/15.3 ± 10.5*	35.8(22.8, 45.7)/30.1(24.4, 35.6)	16.4 ± 11.3/18.3 ± 10.9	27 ± 15/27 ± 13	12(8, 18)/13(8, 21)	10(6, 16)/15(8, 21)	8(2, 15)/13(10, 20)*
TG (mmol/L)	2.16 ± 2.10/1.99 ± 1.23	1.9 ± 1.0/2.1 ± 1.2*	–	1.71 ± 0.99/1.88 ± 1.22*	0.99(0.81, 1.24)/1.47(1.08, 2.24)*	1.45(1.07, 2.05)/1.60(1.18, 2.25)	1.24 ± 0.84/1.54 ± 1.00*	1.5(1.1, 2.2)/1.7(1.2, 2.4)	1.93(1.34, 2.73)/2.12(1.41, 2.95)	1.34(1.03, 1.89)/1.74(1.23, 2.52)
TC (mmol/L)	6.12 ± 1.73/5.43 ± 1.24	4.8 ± 1.1/4.7 ± 1.1	5.08 ± 1.33/5.89 ± 2.60	4.80 ± 1.05/4.74 ± 1.10	4.91 ± 0.94/5.29 ± 1.22*	4.79 ± 1.02/4.78 ± 1.09	4.97 ± 0.98/5.12 ± 1.24*	5.0(3.9, 5.8)/5.4(4.6, 6.4)*	4.83 ± 1.07/4.81 ± 1.17	4.38(3.69, 5.44)/4.99(4.23, 5.97)*
HDL-C (mmol/L)	1.66 ± 0.72/1.34 ± 0.44*	1.3 ± 0.4/1.2 ± 0.4	–	1.28 ± 0.36/1.20 ± 0.35*	1.51 ± 0.45/1.19 ± 0.41*	1.27 ± 0.36/1.20 ± 0.36	1.58 ± 0.49/1.50 ± 0.52*	1.0(0.8, 1.3)/1.1(0.8, 1.5)	1.09 ± 0.29/1.03 ± 0.29	1.10(0.98, 1.34)/1.08(0.88, 1.31)
LDL-C (mmol/L)	3.26 ± 1.19/3.08 ± 1.03	2.7 ± 0.9/2.6 ± 0.9	–	2.75 ± 0.88/2.69 ± 0.89	2.91 ± 0.91/3.26 ± 0.99*	2.77 ± 0.86/2.73 ± 0.88	2.82 ± 0.83/2.95 ± 0.98*	2.8(2.1, 3.4/3.3(2.6, 4.1)*	–	2.74(1.98, 3.24)/2.80(2.30, 3.77)
DR (%)	26/66*	20/31*	50.0/87.9*	18.5/30.5	–	23.51/41.87*	7.8/13.3*	62/69	17.4/29.9	4.4/41.1*
DN (%)	–	–	–	–	–	–	–	–	–	44.4/71.6*
CVD (%)	13/38	31/39*	–	–	19/17	33.54/45.69*	–	–	39.8/48.1*	8.9/7.4

Data are presented as percentages or medians (interquartile range) or mean ± SEM or mean ± SD. *means *P* < 0.05. *NADRI* normoalbuminuric diabetes with renal insufficiency, *ADRI* albuminuric diabetes with renal insufficiency, *N* number, *BMI* body mass index, *HTN* hypertension, *SBP* systolic blood pressure, *DBP* diastolic blood pressure, *HbA1c* glycated hemoglobin, *DD* diabetes duration, *TG* triglycerides, *TC* total cholesterol; *HDL-C* high-density lipoprotein cholesterol, *LDL-C* low-density lipoprotein cholesterol, *DR* diabetic retinopathy, *DN* diabetic neuropathy, *CVD* cardiovascular disease

Compared to patients with micro-ADRI and/or macro-ADRI, the clinical features of NADRI include a higher proportion of females [5, 18, 57], more likely to be older [5, 20] and nonsmokers [18, 23, 58], a shorter diabetes duration [57]; lower levels of systolic blood pressure [20], diastolic blood pressure [20], HbA1c [20, 23, 57], and triglycerides [20]; higher levels of high-density lipoprotein cholesterol[20]; and lower prevalence of hypertension [57], diabetic retinopathy [23, 57], and cardiovascular disease [23] (Table 2).

**Table 2:** Comparison of NADRI with micro-ADRI and macro-ADRI in clinical and laboratory characteristics

Items	MacIsaac 2004 [5]	Rigalleau 2007 [18]	An 2009 [57]	Thomas 2009 [23]	Ekinci 2013 [58]	Vistisen 2019 [20]	
						Type 1	Type 2
*N*	43/38/28	15/36/38	44/50/57	506/295/119	8/6/17	427/264/244	942/664/378
Age (years)	73 ± 1/72 ± 2/67 ± 2*	68 ± 9 /65 ± 11/62 ± 13	59 ± 11 /57 ± 8/61 ± 9	73 ± 1/74 ± 1/71 ± 1	67 ± 2.0/69 ± 2.8/63 ± 2.0	65.0 ± 12.5/59.5 ± 13.4*/48.9 ± 13.2*	70.5 ± 8.5/69.5 ± 9.3*/66.1 ± 10.5*
Female (%)	56/45/18*	66/52/29*	77/66*/42*	64/50/43	62.5/16.7/17.7	62.8/46.2/36.1	52.3/36.0/29.3
BMI (kg/m^2^)	30.8 ± 1.0/29.3 ± 0.7/31.6 ± 1.4	27.0 ± 4.5/27.1 ± 4.5/26.7 ± 4.3	25.2 ± 2.6/23.9 ± 3.0/24.1 ± 2.8	–	34 ± 1.6/33 ± 2.7/31 ± 1.7	25.4 ± 4.6/25.1 ± 3.8/25.4 ± 4.2	30.4 ± 5.6/30.5 ± 5.7/30.7 ± 5.7
Smoking (%)	38/53/62	20/41/63*	18/18/19	5/8*/13*	0/50*/11.8	37.8/46.7/59.6	34.6/39.5/48.6
HTN (%)	-	–	36/52*/89*	–	–	–	–
SBP (mmHg)	138 ± 3/147 ± 3/147 ± 3	143 ± 16/145 ± 19/149 ± 19	–	135 ± 1/134 ± 1/137 ± 2	–	137.8 ± 20.0/138.6 ± 22.5*/144.6 ± 20.3*	136.8 ± 21.0/141.7 ± 21.5*/151.6 ± 23.7*
DBP (mmHg)	75 ± 2/78 ± 1/77 ± 1	79 ± 8/79 ± 9/83 ± 10	–	75 ± 1/75 ± 1/76 ± 1	–	72.8 ± 10.3/74.0 ± 11.3*/80.3 ± 11.4*	73.8 ± 11.2/75.7 ± 11.7*/79.2 ± 12.6*
HbA1c (%)	7.3 ± 0.3/7.9 ± 0.2/7.9 ± 0.3	9.0 ± 1.3/8.6 ± 1.3/8.5 ± 2.0	7.0 ± 1.1/7.9 ± 1.2*/7.2 ± 1.2	7.0 ± 0.1/7.3 ± 0.1*/7.5 ± 0.2*	6.8 ± 0.2/8.0 ± 0.5/7.3 ± 0.4	8.5 ± 1.3/8.8 ± 1.4*/9.2 ± 1.5*	8.1 ± 1.6/8.3 ± 1.7*/8.6 ± 1.7*
DD (years)	14 ± 1/16 ± 1/15 ± 2	14 ± 5/19 ± 12/19 ± 10	5.5 ± 2.3/9.3 ± 3.6*/19.5 ± 5.8*	9 ± 1/11 ± 1/12 ± 1	12 ± 2.4/12 ± 3.5/18 ± 2.1	32.2(22.6, 42.8)/34.6(24.4, 42.6)/29.2(22.6, 37)	12.8(7.6, 18.9)/13.6(7.8, 19.7)/14.5(8.7, 19.6)
TG (mmol/L)	(1.9 X/ ÷ 1.1)/ (1.8 X/ ÷ 1.1)/ (2.0 X/ ÷ 1.1)	2.16 ± 2.10/1.76 ± 0.10/2.21 ± 1.39	1.67 ± 0.94/1.7 6 ± 0.90/1.75 ± 1.01	1.9 ± 0.1/2.0 ± 0.1/2.2 ± 0.1	2.6 ± 0.4/1.8 ± 0.3/2.5 ± 0.4	1.1(0.8, 1.6)/1.2(0.9, 1.8)*/1.5(1.0, 2.2)*	1.8(1.3, 2.7)/2.0(1.4, 3.0)*/2.2(1.6, 3.2)*
TC (mg/dL)	4.4 ± 0.2/4.5 ± 0.2/4.3 ± 0.2	6.13 ± 1.73/5.25 ± 0.98/5.59 ± 1.45	4.42 ± 1.03/4.37 ± 0.91/4.60 ± 0.98	-	4.4 ± 0.2/4.1 ± 0.4/4.7 ± 0.3	5.1 ± 1.1/4.9 ± 1.1/5.5 ± 1.3	4.6 ± 1.2/4.6 ± 1.2/5.1 ± 1.5
HDL-C (mg/dL)	1.15 ± 0.05/1.19 ± 0.06/0.97 ± 0.05	1.66 ± 0.72/1.45 ± 0.49/1.27 ± 0.3*	1.19 ± 0.31/1.16 ± 0.28/1.14 ± 0.26	1.2 ± 0.1/1.3 ± 0.1/1.4 ± 0.1	-	1.8 ± 0.6/1.7 ± 0.6*/1.6 ± 0.6*	1.3 ± 0.4/1.2 ± 0 .4*/1.2 ± 0.4*
LDL-C (mg/dL)	2.6 ± 0.1/2.8 ± 0.1/2.5 ± 0.2	3.26 ± 1.19/2.95 ± 0.83/3.23 ± 1.22	2.46 ± 0.83/2.38 ± 0.85/2.64 ± 0.67	2.4 ± 0.1/2.3 ± 0.1/2.2 ± 0.1	-	2.7 ± 0.9/2.5 ± 0.9/3.1 ± 1.0	2.4 ± 1.0/2.4 ± 1.0/2.8 ± 1.3
DR (%)	26/50/41	26/61*/71*	15/56*/81*	10 ± 1/17 ± 2*/25 ± 4*	50/50/76.5	80/91/96.1	53.6/65.1/74.8
DN (%)	21/27/18	–	–	–	–	–	–
CVD (%)	–	13/31/46	18/20/32	7 ± 1/14 ± 2*/8 ± 2	–	–	–

Data are presented as percentages or medians (interquartile range) or mean ± SEM or mean ± SD or geometric mean X/ ÷ tolerance factor.* means *P* < 0.05 versus the normoalbuminuric diabetes with renal insufficiency. NADRI: normoalbuminuric diabetes with renal insufficiency; micro-ADRI: micro-albuminuric diabetes with renal insufficiency; macro-ADRI: macro-albuminuric diabetes with renal insufficiency; N: number; BMI: body mass index; HTN: hypertension; SBP: systolic blood pressure; DBP: diastolic blood pressure; HbA1c: glycated hemoglobin; DD: diabetes duration; TG: triglycerides; TC: total cholesterol; HDL-C: high-density lipoprotein cholesterol; LDL-C: low-density lipoprotein cholesterol; DR: diabetic retinopathy; DN: diabetic neuropathy; CVD: cardiovascular disease

In addition, compared to patients with normoalbuminuric preserved renal function (eGFR > 60 mL/min/1.73 m^2^), individuals with NADRI are older [16, 28, 59], more frequently females [16, 28, 59] and nonsmokers [16, 28], and have a higher prevalence of hypertension [28, 60] and hyperlipidemia (including triglycerides, total cholesterol, or low-density lipoprotein cholesterol) [28, 59] and metabolic syndrome [59] and cardiovascular disease [24, 28].

## Pathology

A retrospective study found albuminuria or/and low eGFR was absent in nearly 20% [20 of 106] of individuals throughout life showed histopathologic changes characteristic of DKD. Moreover, structural changes were greatly variable, including almost all histopathologic classification of DKD[61].

### Glomerular lesions

A Cohen diabetic rat animal model exhibited extracellular matrix accumulation, mesangial expansion, glomerular basement membrane width increasement, and type IV collagen increasement in the glomeruli, resulting in typical diabetic glomerulosclerosis morphological changes [62]. As early as 1992, Lane et al. reported that the patients with low creatinine clearance rate/normal urinary albumin excretion had more mesangial expansion and glomerular sclerosis compared with patients with normal creatinine clearance rate/normal urinary albumin excretion [9]. Subsequent studies have also shown that patients with NADRI had more advanced glomerular injury compared to individuals with preserved renal function [56, 63].

On the other hand, a significant difference between individuals with NADRI and ADRI was observed, the former had fewer typical glomerular features related with diabetic glomerulopathy or less serious diabetic changes [64, 65]. A previous study found that mesangial area progressively increased from normal control individuals to patients with diabetes and normoalbuminuria, microalbuminuria, and macroalbuminuria, and 3 in 8 patients with NADRI showed typical glomerular changes, whereas the prevalence in patients with microalbuminuria and macroalbuminuria was 5 in 6 and 17 in 17, respectively [58]. Advanced DKD (class III, nodular lesions without global sclerosis in > 50% of the glomeruli or more) was noted in only 27% of patients with NADRI; nevertheless, it was observed in 62% of patients with ADRI [13].

### Tubulointerstitial lesions

Compared to those with normoalbuminuria and preserved eGFR, tubulointerstitial injury of patients with NADRI was more advanced [56, 65]. Similarly, different from typical glomerular changes mostly in individuals with ADRI, predominant interstitial fibrosis and/or tubular atrophy (IFTA) were more frequent findings in patients with NADRI. In the study by Ekinci et al. [58], 3 out of 8 (37.5%) patients with NADRI had disproportionately severe interstitial, tubular lesions; however, only 1 out of 23 (4.3%) individuals with ADRI had these changes.

In addition, in the researches for new biomarkers that identify the risk of reducing glomerular filtration rate (GFR) independent of albuminuria in diabetes, levels of kidney injury molecule-1 (KIM-1), neutrophil gelatinase-associated lipocalin (NGAL), and retinol-binding protein (RBP), tubular damage biomarkers were increased in NADRI, significantly correlated with eGFR and may serve as noninvasive biomarkers of NADRI [66, 67], to some extent, which support the tubular injury in NADRI.

### Vascular lesions

As compared to those with normoalbuminuria and preserved eGFR, vascular lesions in patients with NADRI were more advanced. Likewise, compared to patients with ADRI, vascular lesions of individuals with NADRI were similar or more advanced in contrast to glomerular lesions [56].

Previous clinical observations found, compared to those with or without albuminuria and preserved eGFR, that patients with NADRI are related to a higher risk of major cardiovascular events or cardiovascular morbidity and death [16, 17, 42, 47, 55, 68, 69], and there is a positive association of the prevalence of NADRI with metabolic syndrome and other macrovascular complications [24, 27, 59]. In addition, NADRI shows no or weak relationship with HbA1c and the other major microvascular complications of diabetes (diabetic retinopathy, neuropathy), e.g., up to approximately 30–50% of individuals with decreased eGFR, show neither albuminuria nor retinopathy [22, 24, 28, 36, 37, 57]. Similarly, death in the NADRI is not related to the classic ‘microvascular’ features or factors, such as diabetic retinopathy and glycemic exposure (diabetes duration, HbA1c) [7]. These data indicate that NADRI exhibit distinct clinical characteristics, suggesting predominance of macroangiopathy rather than microangiopathy as underlying renal pathology [5, 24, 65].

Diabetic patients with an eGFR < 60 mL/min per 1.73 m^2^ have a higher renal artery resistance index and a greater degree of intrarenal vascular disease (measured by the intrarenal resistance index) than patients with preserved eGFR, and this difference is independent of UAER[70]. Further studies indicated that patients with NADRI had more serious carotid atherosclerosis [55] and more advanced renal arteriosclerosis than diabetic patients with normoalbuminuria and preserved eGFR [56]. Moreover, in the study by Ekinci et al., in the individuals with NADRI, 7 out of 8 had varying degrees of renal arteriosclerosis [58]. Besides arteriosclerosis, NADRI had more advanced arteriolar hyalinosis compared to patients with normoalbuminuria and preserved eGFR [56].

## Pathogenesis

**Fig. 1:**
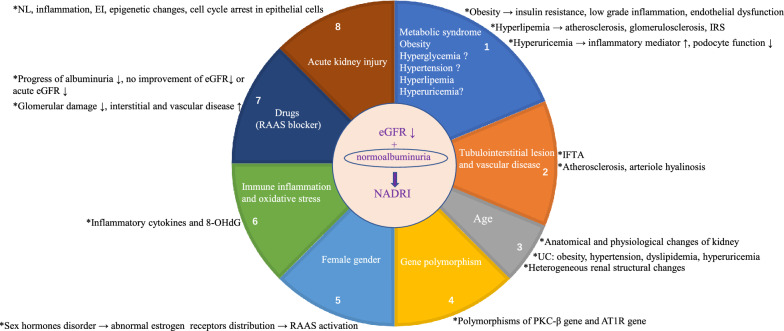
Hypothetical model for the development of NADRI. ? means a point of divergence. *NADRI* normoalbuminuric diabetes with renal insufficiency, *eGFR* estimated glomerular filtration rate, *IRS* insulin resistance syndrome, *IFTA* interstitial fibrosis and tubular atrophy, *UC* underlying conditions, *PKC-β* protein kinase C-β, *AT 1R* angiotensin type 1 receptor, *RAAS* renin-angiotensin-aldosterone system, *8-OHdG* 8-hydroxydeoxyguanosine, *NL* nephron loss, *EI* endothelial injury

Currently, the pathogenesis of NADRI remains to be fully elucidated. The proposed pathogenic mechanisms that underlie NADRI are multifactorial, complex and may involve age-associated renal senescence, hypertension, dyslipidemia, obesity, renal hypertensive and interstitial fibrosis, vascular disease, arteriosclerosis, cholesterol microemboli, lipid toxicity, inflammation, and masking of albuminuria by RAAS inhibitors [4, 5, 22, 28, 58, 70–72]. Based on the clinical research of identified clinical and pathological characteristics, we attempt to propose a hypothetical model for the development of NADRI (Fig. 1).

### Metabolic syndrome

The presence of low eGFR in diabetic patients with normoalbuminuria is associated with the presence of metabolic syndrome [59]. Risk factors that may trigger a progressive decrease of GFR in normoalbuminuria include obesity, hypertension, and high serum triglycerides levels in diabetes [73], and these mechanisms often play a role in nephrosclerosis [74].

#### Obesity

In T2DM, obesity from the overweight category (body mass index 25–29.9 kg/m^2^) to severe obesity (body mass index > 35 kg/m^2^) was related to reduced renal function in the absence of albuminuria [75]. Increased waist circumference was independent risk factor for renal insufficiency[29].

Adverse effects of obesity may include insulin resistance, low-grade inflammation, and endothelial dysfunction, and these pathways have been reported as potential mechanisms for the development of renal insufficiency [76–78].

#### Hyperglycemia

The Atherosclerosis Risk in Communities Study, which enrolled 1871 patients with diabetes, showed a similar decline in the incidence of decreased eGFR < 60 mL/min/1.73 m^2^ for every 1% reduction in HbA1c, regardless of preexisting albuminuria [79]. Poor glycemic control is an independent predictor of progression to end-stage renal disease in T1DM patients with normoalbuminuria [80]. These data indicated that hyperglycemia may be associated with eGFR decline in NADRI.

However, HbA1c is an independent risk factor for albuminuria, but not for GFR impairment [24, 29, 75]; thus, NADRI is probably less related to hyperglycemia. Some researches propose that diabetes contributes to the NADRI development via pathways which may be partially separated from hyperglycemia. Amy et al.’s study showed that in the diabetic patients with low and even ‘normal’ HbA1c levels, the prevalence of NADRI is still higher than that in the nondiabetic individuals [2]. NADRI is more common in individuals with good glycemic control [55]. Moreover, both HbA1c levels and the prevalence of microvascular complications are lower in individuals with NADRI [5, 18, 22, 23, 28]. These data indicate that potential pathogenic mechanisms of NADRI may be at least partially independent of hyperglycemia [2].

#### Hypertension

Among normoalbuminuric diabetes, higher prevalence of hypertension is observed in patients with low eGFR than in those with preserved eGFR. Blood pressure perturbations may contribute to reduce kidney function independently of albuminuria [58]. Each 10 mmHg increase in mean systolic blood pressure was related to a 15% increase in the hazard ratio for renal insufficiency defined as eGFR < 60 mL/min/ 1.73m^2^ or doubling blood creatinine level[29]. These studies provided clinical evidence that hypertension may contribute to renal insufficiency and play a role in the development of NADRI.

However, in the study by Mottl et al. [2], the lower prevalence of NADRI in individuals with poor control of hypertension suggested hypertension was less likely to be a key factor of NADRI. In addition, there is a well-documented relationship between hypertension and albuminuria in diabetes [81]. On the other hand, it cannot exclude the threshold effect of blood pressure on albuminuria and/or on renal damage mediated by hypertension [2]. Therefore, underlying pathogenesis of NADRI may be at least partially independent of hypertension.

#### Hyperlipidemia

Low eGFR in normoalbuminuric diabetes was related to high levels of triglycerides [59]. Hyperlipidemia (high triglycerides, high total cholesterol, or low high-density lipoprotein cholesterol) is the independently factor related to renal impairment [28, 75, 82]. A recent study has reported that increased lipid abnormalities play a certain role in the occurrence and development of NADRI [71]. Further studies indicate that insulin resistance syndrome may underlie or mediate the relationship between lipids and renal function loss [82] and hypercholesterolemia may cause kidney damage by atherosclerosis and glomerulosclerosis [83].

In addition, treatment of dyslipidemia may reduce the incidence of early renal disease [82], and the relationship between cholesterol-lowering treatment and renal dysfunction progression in diabetes was independent of UAER [84].

#### Hyperuricemia

Previous studies have reported an independent association between serum uric acid levels and eGFR decline in diabetes [85–89]. A large study of 1052 cases showed that serum uric acid may play an important role in the reduction of eGFR in elderly diabetic individuals with normoalbuminuria [90]. Serum uric acid-promoting eGFR loss may be due to proinflammatory mechanisms [91] and led to renal fibrosis, glomerulosclerosis, and tubular lesions with inflammatory mediators elevation and decreased podocyte function [92]. However, Hanai et al. showed that high serum uric acid level was a prognostic factor for renal function decrease only in individuals with preserved renal function [93].

Currently, a randomized, double-blind, placebo-controlled, multicenter clinical trial that has enrolled 530 patients with T1DM, the Preventing Early Renal Loss in Diabetes Study, will explore whether the effect of lowering serum uric acid with allopurinol should be able to slow GFR loss on mild to moderate DKD with or without albuminuria. In this study, nearly one-fifth of normoalbuminuric patients with declining kidney function were enrolled, and the results of the clinical trial are worth looking forward to [94].

### Tubulointerstitial lesion and vascular disease

Clinical pathological studies have demonstrated that predominant tubulointerstitial and vascular lesions are more frequently observed in NADRI. In diabetes, tubulointerstitial injury (e.g., interstitial fibrosis, interstitial fibrosis and tubular atrophy) [13, 56, 61, 95] and vascular lesions (e.g., intrarenal vascular disease) are the factors associated with low eGFR, independently of albuminuria [56, 70]. It is speculated that declining of eGFR may be partly related to tubulointerstitial and vascular lesions in NADRI.

The markers of renal tubular injury, such as NGAL, RBP, and KIM-1, have been found significantly increased in NADRI and weakly but significantly correlated with eGFR, suggesting that renal tubular interstitial injury may be associated with the development of NADRI [66, 96].

Further studies show that the decline in GFR in T2DM with or without microalbuminuria is associated with an increase in carotid intimal-medial thickness, carotid stiffness, and intrarenal arterial resistance index [97], which suggest that the reduction in GFR is partly due to generalized increase in arteriosclerosis [70]. Subsequent studies showed that arteriosclerosis may contribute to decrease in renal function independently of albuminuria and may be playing pathogenic roles [58]. Arteriosclerosis is the underlying mechanisms of glomerulosclerosis [98], and diabetic patients with glomerulosclerosis present with a more rapid decline in eGFR [99]. The main renal arteries stenosis caused by atherosclerosis increases the susceptibility to kidney ischemia [100]. A proportion of patients with rapid decline of eGFR exhibited normoalbuminuria, which may be associated with ischemic kidney changes caused by the intrarenal arteries atherosclerosis [101, 102].

Moreover, interstitial fibrosis, tubular atrophy, and arteriosclerosis in renal biopsy specimens are the pathological factors to develop kidney events [56]. Besides arteriosclerosis, arteriolar hyalinosis is related to global glomerular sclerosis because it reduces the glomerular blood flow [103] and has been found to be a histological predictor for GFR decline in diabetic patients with normoalbuminuria [104].

### Age

Some clinical studies of NADRI show that patients with NADRI are more likely to be older, and age is an independent risk factors for renal insufficiency in T2DM patients with normoalbuminuria [55, 66]. This fact should probably indicate that age may play a role in the development of NADRI.

Aging can lead to various anatomical and physiological changes of the kidney, including structural changes of aging with age, declining GFR, and decreased tubular function [105, 106]. In addition, elderly diabetic patients usually have increased underlying conditions, such as obesity, hypertension, dyslipidemia, and hyperuricemia, which may result in renal function loss and nephrosclerosis via arteriosclerosis.

More significant heterogeneity in renal structural changes of NADRI (atypical tubulointerstitial and vascular lesions) than in ADRI suggested aging may be one of the key contributors to the atypical nephropathy changes observed in NADRI and contributes to decline in kidney function independently of albuminuria [58]. In particular, hyaline arteriolosclerosis is a characteristic of ‘age-related vasculopathy’ and is related to global glomerular sclerosis [107], which is a histological predictor for GFR decline in normoalbuminuric patients with T2DM [104].

### Gene polymorphism

The prevalence of NADRI varies by ethnic groups, indicating the development of the disease may involve genetic susceptibility. Polymorphism of the protein kinase C-β gene was related to accelerated decline of eGFR in T2DM without overt proteinuria, and the T-G haplotype of protein kinase C-β gene (PRKCB1) may be a valuable predictive factor for the rapid deterioration of kidney function [108].

In addition, the relationship between polymorphism of angiotensin type 1 receptor gene and normoalbuminuric chronic kidney disease among South Indian T2DM patients was described. Compared to ADRI, the relative risk of AC genotype and C allele in NADRI was 4 times higher. Significant correlation of AGTR1 A1166C polymorphism was noted in NADRI as compared to ADRI [109].

### Female gender

The effects of gender on renal injury are controversial. Lotfinejad et al. noted renal damage in diabetes was directly related to male gender, but not to female gender [110]. In vitro experiment data showed 17β-estradiol can attenuate glomerulosclerosis and renal tubulointerstitial fibrosis in streptozotocin-induced diabetes mellitus [111]. However, a study on obese mice with T2DM found that female mice had renal injury earlier than male mice [112]. Al-Trad et al. [113] suggested that diabetes may give rise to sex hormones disorder and affected estrogen receptors distribution in the renal tissues; as a result, estrogen had no protective effect on the kidney in diabetic patients but aggravated the decline in GFR. Estrogens play a part in regulating and responding to the components of RAAS [114], activation of which is harmful to renal function [115].

Epidemiological studies show that the prevalence of NADRI is higher in female gender. The female predominance may be due to sex differences in CKD risk [116]. Female gender is an independent risk factors for renal impairment [29, 36, 75, 117]. Previous study described that decreased GFR in eight normoalbuminuric long-standing T1DM women was related to worse diabetic glomerular injury [9]. Tsalamandris et al. also well described a relatively small group of normoalbuminuric long-standing diabetic largely female individuals with declined GFR [21]. These facts indicate that reduced GFR is much more common among female patients [63].

Subsequent studies have demonstrated women with diabetes are at greater risk than men for accelerated GFR decline in the absence of albuminuria [28]. This effect is best shown by UKPDS-7426 study [29], which found that during a follow-up period of up to 15 years, women were at increased risk.

### Immune inflammation and oxidative stress

Tumor necrosis factor alpha (TNF-α), interferon γ, interleukin (IL)-10, IL-6, KIM-1, and other inflammatory cytokines are involved in the immune inflammatory response of DKD [96, 118]. Further studies have shown proinflammatory interleukins are related to podocyte lesions and proximal tubular dysfunction in early DKD, which could play a key role in the pathogenesis of early DKD, prior to the development of albuminuria [119]. Inflammatory cytokines were associated with early kidney damage and predict progression in patients without albuminuria [120].

The concentration of serum Fas-pathways cytokines (soluble Fas and Fas ligand) and TNF-α (soluble TNF receptor 1, soluble TNF receptor 2) are related to reduced eGFR independent of urinary albumin excretion in diabetes [66, 121–123]. Li et al.’s study found that TNF-α was independent risk factors for normoalbuminuric kidney insufficiency in T2DM [66]. Further studies show TNF-α can directly decrease glomerular blood flow and increase glomerular vasoconstriction, leading to the decline of glomerular filtration rate [124].

In addition, serum IL-17A, IL-18, and macrophage inflammatory protein 1 alpha were predictors of NADRI [125, 126]. Urinary inflammatory cytokines, IL-1, IL-6, IL-10, TNF-α, and interferon-γ, especially IL-6 and IL-10, may assist in the identification of DKD in T2DM patients, even in the absence of micro- and macroalbuminuria [127]. Consequently, immune inflammation plays an important role in the decline of renal function of NADRI.

Besides immune inflammation, oxidative stress may contribute to the development of NADRI. 8-hydroxydeoxyguanosine, as a sensitive marker of intracellular oxidative stress, is more highly expressed in high intima media thickness than in normal intima media thickness and is positively related to coronary heart disease risk scores [128]. The clinical features and renal histopathologic changes study of NADRI showed that macroangiopathy was more prevalent and often accompanied by cardiovascular disease. What is more, 8-hydroxydeoxyguanosine is an independent risk factor for NADRI [66].

In addition, urinary NGAL and RBP are biomarkers for NADRI, which may be caused by inflammation and oxidative stress and lead to tubular damage [66, 129].

### Drugs

For the past decades, the use of RAAS blockers has increased [50] and recent studies report that the values are up to 70% or more [24, 36, 37, 47]. What is more, the use of RAAS blockers and prevalence of NADRI have parallelly increased. Although significant difference was not observed after ruling out the effect of RAAS blockers, the prevalence of NADRI was generally lower than the use of RAAS blockers was introduced [5]. Previous studies have shown that the use of RAAS blockers in diabetes can prevent the development of microalbuminuria[130, 131]. In a study of the renal structure of NADRI, 2 in 10 patients with normoalbuminuria developed microalbuminuria at 4–6 weeks after the cessation of RAAS inhibitor treatment [58]. Further studies indicate that the occurrence of NADRI is closely associated with the application of RAAS blockers [55]. Therefore, the therapeutic effect of RAAS blockers may take a part in the pathogenesis of NADRI.

At present, there are two hypotheses on the pathogenesis. One is that the application of RAAS blockers slows the progression of albuminuria in diabetes, while the decline in eGFR is not significantly improved, even possibly resulting in an acute decline of eGFR [50, 51, 55, 132]. As far as purely theoretical grounds, under certain circumstances, RAAS blockers may be deleterious, because these agents increase the susceptibility to renal ischemia due to certain causes (atherosclerosis, sepsis, immunosuppressants, etc.) via preventing the increase of efferent arteriolar resistance [100].

The other proposed mechanism is that the use of RAAS blockers in diabetes may result in a decline in the prevalence of glomerular damage and allowed the occurrence of other renal injury, such as interstitium and vascular damage, which may lead to progressive renal injury without triggering albuminuria [74].

### Acute kidney injury

In diabetes, due to various factors, such as ischemia, infection, nephrotoxicity, or obstruction, acute kidney injury (AKI) often occurs in a mild and unrecognizable form, resulting in progressive decline in kidney function independent of albuminuria [133]. Onuigbo et al. also reported that once AKI occurs in diabetic patients, renal function decrease is more likely, and this is independent of albuminuria [134]. Single or recurrent AKI remarkably increases the risk of advanced CKD, and AKI is a risk factor for CKD independent of albuminuria in diabetes [135].

Several mechanisms have been proposed for the progression of AKI to CKD, such as nephron loss, inflammation, endothelial injury with vascular rarefaction and hypoxia, epigenetic changes, and cell cycle arrest in epithelial cells, ultimately leading to progressive glomerulosclerosis and tubulointerstitial fibrosis [136–139].

## Diagnosis

**Fig. 2:**
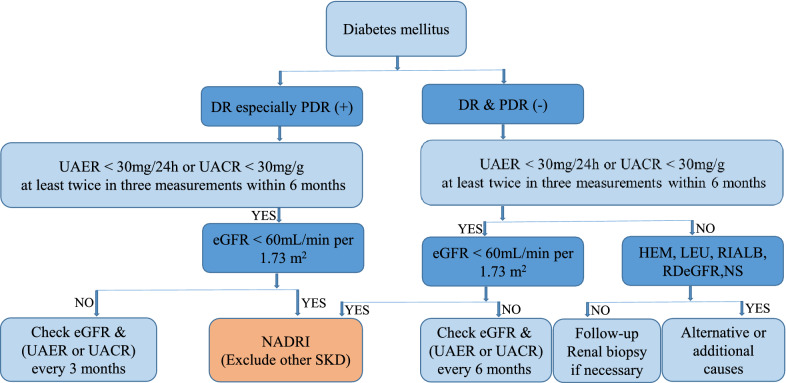
Flow chart for diagnosing NADRI. *DR* diabetic retinopathy, *PDR* proliferative diabetic retinopathy, *UAER* urinary albumin excretion rate, *UACR* urinary albumin creatinine ratio, *eGFR* estimated glomerular filtration rate, *HEM* hematuria; *LEU* leukocyturia, *RIALB* rapidly increasing albuminuria, *RDeGFR* rapidly decreasing estimated glomerular filtration rate, *NS* nephrotic syndrome, *NADRI* normoalbuminuric diabetes with renal insufficiency, *SKD* secondary kidney diseases

Kidney Disease Outcome Quality Initiative (KDOQI) guidelines recommend that the diagnosis of DKD should be based on monitoring UACR or UAER, fundus changes, and evaluation of eGFR. Based on KDOQI guidelines recommendations and clinical studies of NADRI, we attempt to propose the diagnostic criteria of NADRI: (1) to meet the latest diabetes diagnostic standards of World Health Organization or American Diabetes Association (ADA); (2) to test and prove within 6 months, UAER < 30 mg/24 h or UAER < 20 µg/min or UACR < 30 mg/g, at least twice in three measurements, to avoid confounding by transient increases in conditions, such as exercise, fever, hematuria, urinary tract infection, and congestive heart failure[140]; (3) to detect within 6 months, eGFR < 60 mL/min/ 1.73 m^2^, at least twice in three measurements, and exclude eGFR reduction caused by AKI and other reasons; and (4) to exclude other secondary nondiabetic kidney diseases (Fig. 2).

eGFR estimation formula based on serum creatinine is generally recommended for diagnosis of DKD, including Cockcroft-Gault formula, modification of diet in renal disease formula, and CKD epidemiology collaboration (CKD-EPI) formula [141, 142]. However, serum creatinine level is susceptible to be influenced by factors, like muscle mass, dietary protein intake, hyperlipidemia, hemolysis, etc.; the sensitivity in eGFR is insufficient. Therefore, in recent years, the Kidney Disease: Improving Global Outcomes (KDIGO) and ADA proposed to use the eGFR calculation formula based on serum creatinine and cystatin C, such as CKD-EPI cystatin C formula and CKD-EPI creatinine-cystatin C formula [143]. Consequently, according to the latest KDIGO guidelines, we recommend adding the detection of serum cystatin C, and the eGFR may be calculated according to the cystatin C and creatinine-cystatin C formula.

## Conclusion

Epidemiological data demonstrate that NADRI is a prevalent clinical phenotype of DKD. Moreover, NADRI exhibits distinct clinical characteristics and a wide heterogeneity of histology features. Based on the studies of clinical features and renal histopathologic changes of NADRI, we attempt to propose an underlying pathogenesis model and a flow chart for diagnosing NADRI, which increases the practicality and reliability of the study. The underlying pathogenesis associated with NADRI involves multiple factors and comprises the usual suspects, including obesity, hyperlipidemia, tubulointerstitial lesion and vascular disease, age, gene polymorphism, female gender, immune inflammation and oxidative stress, drugs, and AKI, while appears to be at least partially independent of hyperglycemia and hypertension and does not exclude the possible role of hyperuricemia. Early diagnosis and a deeper understanding of pathogenesis may provide theoretical guidance for the therapy of NADRI and prevent or delay the CKD progression, end-stage renal disease, and diabetes complications as far as possible.

Although many of factors are discussed in this review, the list is not exhaustive. Multilevel, multicenter, large-sample clinical studies are needed to further clarify the pathogenesis of the disease, discover specific biomarkers, establish clear and precise laboratory diagnostic standards for the disease, and provide an important theoretical basis for preventing the occurrence, development, early intervention, and treatment of the disease.

## Data Availability

All data were collected from the publication articles.

## Abbreviations

DKD: Diabetic kidney disease
eGFR: Estimated glomerular filtration rate
UAER: Urinary albumin excretion rate
UACR: Urinary albumin creatinine ratio
NADRI: Normoalbuminuric diabetes with renal insufficiency
CKD: Chronic kidney disease
ADRI: Albuminuric diabetes with renal insufficiency
T1DM: Type 1 diabetes mellitus
T2DM: Type 2 diabetes mellitus
RAAS: Renin-angiotensin-aldosterone system
HbA1c: Hemoglobin A1c
GFR: Glomerular filtration rate
IL: Interleukin
IFTA: Interstitial fibrosis and tubular atrophy
KIM-1: Kidney injury molecule-1
NGAL: Neutrophil gelatinase-associated lipocalin
RBP: Retinol-binding protein
AKI: Acute kidney injury
KDOQI: Kidney Disease Outcome Quality Initiative
ADA: American Diabetes Association
KDIGO: Kidney Disease: Improving Global Outcomes
